# Serendipity in oto-rhino-laryngology

**DOI:** 10.1007/s00405-024-08475-6

**Published:** 2024-02-07

**Authors:** Pek van Andel, Louw Feenstra, Henk G. Schmidt

**Affiliations:** 1 Groningen University, Groningen, The Netherlands; 2 Erasmus University Rotterdam, Rotterdam, The Netherlands

**Keywords:** Serendipity, Unsought findings, Oto-rhino-laryngology

## Abstract

**Introduction:**

Serendipitous findings are findings that were initially unsought but nevertheless contribute to the development of the discipline. This article reviews eight serendipitous findings in oto-rhino-laryngology important to its advancement.

**Method:**

The following serendipitous findings are discussed: the accidental discovery of the laryngeal mirror and indirect laryngoscopy by Garcia (1854), the invention of direct oesophagoscopy by Kußmaul (circa 1868), Czermák’s (1863) development of diaphanoscopy, the unintentional emergence of bronchography from a clinical error made by Weingartner (1914), adenotomy by Meyer (1869), the discovery of the causes of unbalance related to the vestibular nerve by Flourens (1830), Bárány’s (1914) finding that the semi-circular canal reflex is involved in equilibrium, and the relationship between gastroesophageal reflux and middle-ear infections by Poelmans and Feenstra (2002).

**Discussion:**

Based on these case studies we conclude that serendipity, defined as the art of making an initially unsought find, does not always appear out of nowhere. Often the researcher is already wrestling with a problem for which the serendipitous finding provides a solution. Sometimes the serendipitous finding enables the application of a known solution to a new problem. And sometimes a serendipitous finding is not recognized as such or considered unimportant. Since observations tend to be theory-loaded, having appropriate background knowledge is a *conditio sine qua non* to elaborate an unanticipated observation.

New medical observations are generally made by chance (…) The initiative of the physician consists in seeing and not let slip that opportunity presented to him, and his only merit is to observe precisely (…) There is nothing accidental, and what is for us accident is only an unknown fact that may become, if explained, the occasion for a more or less important discovery.^1^

Claude Bernard

(…) (by chance you might say, but remember that in the observational sciences chance favours only prepared minds.)^2^

Louis Pasteur

The most exciting thing you will ever hear in a laboratory is not “Eureka!” but, “Hmm, that’s funny”.

Attributed to Isaac Asimov

## Introduction

This view of science is prevalent in the scientific community: that scientific endeavour is a rational and deliberative activity. Its purpose is the creation of theories on how the world works. The scientist proposes new hypotheses based on such theory and collects empirical data to test them. The results of such tests can contribute to the growth of our knowledge of the world. This view of science ignores, however, the importance of unplanned and unexpected discoveries, findings that are not necessarily in line with prevailing theory and seem to drop out of the sky. A well-known example is the discovery by John Snow of the sources of cholera in 1850 London, a finding that contributed to the demise of the then prevailing miasma theory attributing cholera and the plague to ‘bad air.’ The discovery of unanticipated phenomena is called serendipity. Serendipity can thus be defined as the art of making an initially unsought finding [3].

The purpose of the present contribution is to bring together for the first time important serendipitous discoveries in the field of oto-rhino-laryngology (ORL).^3^ First, we will make a few brief remarks on the history of the serendipity construct. Subsequently, eight discoveries contributing to knowledge and techniques supporting the maturation of ORL as a science will be presented in the form of short vignettes. Finally, we will critically discuss the issue of serendipity in the light of the examples provided: To what extent are serendipitous findings made by chance?

### History of serendipity

The story goes that it was Horace Walpole, the eighteenth-century man of letters who coined the term, drawing gradual attention to the serendipity phenomenon. Walpole derived the word “serendipity” from the title of a French version of the sixteenth-century Italian version of the old Persian fairy tale of Amir Khusrau (1302) about ‘The peregrination of the three Princes of Serendip’. “As their Highnesses travelled,” Walpole remarks, “they were always making discoveries, by accidents and sagacity, of things which they were not in quest of” ([5], p. 238). Walpole’s letter was virtually forgotten but revitalized by the polymath and sociologist of science Robert K. Merton, who came across the term in the Oxford English Dictionary [6].

The historian of science Sean Silver, however, makes short thrift with this origin story. He argues that the concept itself, specifically its critical pairing of “sagacity” and “accident”—pays much older debts. It goes back to Francis Bacon who interpreted the old Greek fable of Pan finding Ceres as a metaphor for finding what was not sought after. While the other gods set out to find Ceres, who had absented herself from the Olympus due to grief, Pan preferred to go hunting. And while he was pursuing game, he accidentally found Ceres. Bacon concludes that it was Pan’s sagacious experience and general knowledge of nature that enabled the discovery of Ceres “whilst the pursuit was directed another way” ([5], p. 243).

In academic literature serendipity is usually defined as the art of making a surprising observation, followed by a correct abduction, to explain the observation. Aristotle already gave an interesting example: the surprising observation that the moon presents itself with different faces. The correct abduction is that all these faces consist of sunlight, reflected by the moon. The emergence of serendipity is, however, not yet well understood. To what extent is the scientist, the artist, the inventor, cognitively prepared to recognize an unanticipated finding as relevant for the problems he or she faces?

Hans Reichenbach (1891–1953), philosopher of science, makes a distinction between the context of discovery and the context of justification. The way theories are justified is rule-based and subject to restrictions. The context of discovery is, however, free of rules [7]. Karl Popper (1902–1994) argues that scientists may draw upon diverse sources of inspiration, such as metaphysical beliefs, dreams, religious teachings, or everyday experiences. The serendipitous discovery clearly belongs to this category. How theories are conceived is not the business of philosophy of science but the realm of cognitive psychology [8].

## Serendipitous discoveries in ORL

In this section we will describe eight serendipitous discoveries that contributed to the development of ORL. We will do this in the form of brief vignettes that try to encapsulate both the discovery and its context.

### The discovery of the laryngeal mirror and indirect laryngoscopy

One afternoon in September 1854, Manuel *Patricio Rodríguez* García (1805–1906), a Spanish baritone, music educator, and former professor of singing at the Paris Conservatoire, was strolling in the garden of the Palais-Royal, now The Louvre, and observed the flashing of the sun in the windowpanes of the seventeenth-century quadrangle. Prompted by Felix Semon, he told the story in the Transactions of the Section of Laryngology of the International Congress of Medicine in London, in 1881: “(…) preoccupied with the ever-recurring wish so often repressed as unrealizable, suddenly I saw the two mirrors of the laryngoscope in their respective positions, as if actually present before my eyes. I went straight to Charrière, the surgical instrument maker and asking if he happened to possess a small mirror with a long handle, was informed that he had a little dentist's mirror, which had been one of the failures of the London Exhibition of 1851. I bought it for six francs. Having also obtained a hand mirror I returned home at once, very impatient to begin my experiments. I placed against the uvula the little mirror (which I heated in warm water and carefully dried): then, flashing upon its surface with the hand mirror a ray of sunlight, I saw at once, to my great joy, the glottis wide open before me, and so fully exposed that I could perceive a portion of the trachea. When my excitement had somewhat subsided, I began to examine what was passing before my eyes. The manner in which the glottis silently opened and shut, and moved in the act of phonation, filled me with wonder” ([9], pp. 197–198).

### The discovery of the direct oesophagoscopy

In 1868 in Freiburg, north of Basel, a sword-swallower performed his act in a tavern, the *Wirtshaus Wolfschlucht.* Dr. Keller, a senior house officer, was fascinated and invited the sword-swallower to have his throat examined, which was performed by Dr. Muller, a registrar. Dr. Muller suggested to Professor Carl Philipp Adolf Konrad Kußmaul (1822–1902) to examine the sword-swallower’s gullet. Kußmaul, specialist in internal medicine, took a special interest in the position of the head and neck in relation to the body during the act of swallowing the blade. Kussmaul decided to investigate the sword-swallower with an instrument invented by Antonin Jean Desormeaux (1815–1894). Desormeaux’s endoscope was constructed in Paris in 1853, to be used as an instrument for endoscopy of the urethra and bladder after the example of Philipp Bozzini (1773–1809), the first to construct a specific instrument intended to be used in gynaecology and urology.

Kußmaul asked the instrument maker in town to make two tubes—one round and one oval—of 47 cm length and 13 mm cross-section. The sword-swallower tolerated the long tubes well. But, the investigation was disappointing because the light source was not strong enough to illuminate a narrow field so distant. The light source was subsequently improved, and several details of the oesophagus investigated. Kußmaul and the sword-swallower demonstrated the straight oesophagoscope in different clinics. Kußmaul was criticized, mainly by the French medical press. It was said that it was easy to bring a tube in a quack, as one critic called the sword-swallower, but impossible with patients not used to swallowing swords. It was, however, the sword-swallower who showed the way to stretch the angle between the mouth and the throat. The sword served as a model for the future oesophagoscope [10, 11].

### Diaphanoscopy

Transillumination started by using sunlight to illuminate the larynx through the skin from the front of the neck. The overlying skin, subcutis and cartilage of the larynx and trachea are sufficiently translucent to permit indirect laryngoscopy with the Garcian-mirror [10]. To obtain optimal results, though, a dark room is preferred. Johann Nepomuk Czermák (1828–1870) therefore concluded that for rhinoscopy transillumination was less satisfactory [12]. Friedrich Eduard Rudolph Voltolini (1819–1889) in Breslau picked up Czermák’s diaphanoscopy and applied it to the nose. As soon as Thomas Alva Edison and Joseph Swan in England simultaneous invented the carbon filament light, Voltolini, using such light, applied diaphanoscopy to the paranasal sinuses [10].

### Bronchography

Max Weingartner (1882–?) of Berlin was using a bismuth solution during an X-ray examination of a patient with an oesophageal cancer when some of the swallowed solution accidentally spilled into the lungs. In that way, bronchography was discovered. The finding was taken up by Chevalier Jackson in 1918. And lipiodol was introduced as a contrast medium in 1922 [13].

### Adenotomy

Hans Wilhelm Meyer’s (1824–1895) finger made him accidentally aware of the presence of what he described as ‘a morbid growth’ in the nasopharynx. He first described the adenoid in 1869 and his paper On adenoid vegetations in the nasopharyngeal cavity was translated into several languages. He recognized the condition as quite common, as he found 102 cases in eighteen months. The paper was so detailed that even someone inexperienced in posterior rhinoscopy could suspect and detect the presence of adenoid hypertrophy with ease. Meyer recommended the removal of the adenoid with the aid of a ‘ring-knife.’ [14].

### Disorders of balance and the vestibular nerve

Marie Jean Pierre Flourens (1794–1867) operated on pigeons. He noticed that severing of the semi-circular canals led to a change of posture and abnormal movements of the head. He realized that the acoustic nerve consisted of two parts, a cochlear part for hearing and a vestibular part, associated with the semi-circular canals, for equilibrium [13, 15]. These findings were later verified by Josef Breuer (1842–1925) of Vienna, who in 1874, published his results of balance experiments in animals and was the first to note that the phenomenon of nystagmus was a labyrinthine reflex.

### A reflex of the semi-circular canals

The young otologist, Robert Bárány (1876–1936) worked in Professor Politzer's Clinic in Vienna. He writes: “Among my patients there were many who required syringing of the ears. A number of them complained afterwards of vertigo. Obviously, I examined their eyes and I noticed in doing this that there was nystagmus in a certain direction. I made note of this. After a time, when I had collected about twenty of these observations, I compared them one with another and was amazed always to find the same note. I then realized that some general principle must be implied, but at the same time I did not understand it. Chance came to my aid (Italics by us). One of my patients, whose ears I was syringing, said to me: “Doctor, I only get giddy when the water is not warm enough. When I do my own ears at home and use water warm enough, I never get giddy”. I then called the nurse and asked her to get me warmer water for the syringe. She maintained that it was already warm enough. I replied that if the patient found it too cold, we should conform to his wish. The next time she brought me very hot water in the bowl. When I syringed the patient's ear he shouted: “But Doctor, this water is much too hot and now I am giddy again”. I quickly observed his eyes and noticed that the nystagmus was in an exactly opposite direction from the previous one when cold water had been used. It came to me in a flash that obviously the temperature of the water was responsible for the nystagmus. From this, I immediately drew certain conclusions. If the temperature of the water was really responsible, then water at exactly body temperature should cause neither nystagmus nor vertigo. An experiment confirmed this conclusion. Furthermore, I said to myself, if it is the temperature of the water, nystagmus must be caused in normal cases also and not only in cases of suppurating ears. This I was also able to prove” [16].

Robert Bárány received the 1914 Nobel Prize in Physiology or Medicine for his work on the physiology and pathology of the vestibular apparatus.

### Otology and gastroesophageal reflux

A 72-year-old patient was referred for a second opinion to the second author. The patient complained of a running left ear of 6 months duration. He had never suffered from any ear problems before. His ailment had so far been resistant to treatment by his general physician and his first puzzled ear-surgeon. Whatever was examined or done, no causal explanation was found. The patient used for many years heartburn medication (omeprazol). Because his pharyngitis advanced and moreover a posterior laryngitis was diagnosed his medication was doubled. That worked well for his sore throat; moreover, his left ear went back to normal within a couple of weeks. This chance finding led to the speculation that the patient’s gastroesophageal reflux had led to (pan)pharyngitis, subsequently tubotympanitis and (sub)chronic otitis [17]. Further methodical clinical research found this phenomenon in many more patients and demonstrated that this hypothesis appeared indeed to be correct [18].

## Discussion

The serendipitous finding is often considered as an enigma. It occurs seemingly out of nowhere, discovered purely by chance. Its emergence strikes as accidental and cannot be predicted. Discovering relevant new empirical facts appears to be a lucky draw. However, the review of the serendipitous findings from the domain of ORL conducted above suggests at least some conditions under which serendipity may occur. We will discuss here four of these conditions: (1) the emergence of a serendipitous finding in response to a problem perceived by the scientist, (2) the application of a known solution to a new problem, (3) the context in which a serendipitous occurs, and (4) the role of background knowledge.

### Serendipity in response to a perceived problem

Attempts to study the cavities of the human body already had a long history before Garcia invented the laryngeal mirror^4^. According to Lapeña [19] his predecessors from Aranzi (studying the nasal cavity) to Bozzini wrestled with how to focus light to unearth what previously was unseen. Bozzini (1806) states that “if a person wishes to see around a corner … the rays must be broken, and a mirror is required for illumination and reflexion” ([20], p. 605). It was this problem that clearly was on Garcia’s mind while trying to find a way to observe the larynx. He states that while walking in the parc of the Palais Royal and while seeing the sunlight flashing on the windows he was “(…) preoccupied with the ever-recurring wish so often repressed as unrealizable, (and) suddenly (…) saw the two mirrors of the laryngoscope in their respective positions…” ([9], pp. 197) Bárány’s finding that the semi-circular canal reflex is involved in equilibrium, showed that same pattern. While syringing the ears of some of his patients, he noted the vertigo and nystagmus problems. The solution presented itself by the accidental discovery that the water temperature was critical [16]. Their serendipitous findings were not coming out of thin air but were an original response to a problem that already bothered them.

### Serendipity as the application of known solution to new problem

Kußmaul was specialist in internal medicine, in the nineteen century an emerging discipline lacking opportunities to examine the internal organs directly. The endoscope, in use for endoscopy of the urethra and bladder was already invented by Desormeaux [10]. The accidental confrontation with the sword swallower by his senior house officer Keller made Kußmaul aware that he could use a similar technique in the examination of the oesophagus. Czermák’s invention of diaphanoscopy [12] and its further development by Voltolini are probably additional examples of serendipity as the application of an already-known solution to a new problem [10].

### ‘Negative serendipity’

A serendipitous finding is sometimes made while the time does not yet seem ripe or because the discovery is not taken seriously. As a result, the finding is lost. The case of the floppy-eared rabbit is instructive here. Two researchers discovered independently and by accident the same phenomenon: reversible collapse of rabbits' ears after injection of the enzyme papain. One of them published the phenomenon as a serendipitous find, whereas the other took the finding not as serious as other aspects of his experiments involving the injection of papain. The latter thought it was a funny but not very relevant by-product of his scientific work [21].

As a young medical student, the first author learned that situs inversus stands for a mirror position of the intestines, heart, and lungs. An example of such a phenomenon is the Kartagener syndrome consisting of complete situs inversus, bronchiectasis, and chronic sinusitis. He saw an illustration of the beating cilia cleaning the sinus by moving the mucus as a vortex, a spiral towards the exit to the nasal cavity. That gave him the hunch that the Kartagener’s cilia might beat ‘inverted’, i.e., in the wrong direction which might be the root of the problem. With the associate professor in ORL he examined under a microscope the biopsies of nasal mucosal linings of several Kartagener syndrome cases. Surprisingly, none of them showed beating cilia. So, his hunch proved to be wrong. However, even the consistent observations of silent cilia of all mucosal specimens of the Kartagener syndrome patients were not a stimulus to publication in those days. The disappointed student was advised to continue his studies. Some years later the immobile cilia and their deviant microstructural skeletons were discovered by Afzelius, and the Kartagener Syndrome was renamed as ‘primary immobile ciliary syndrome’ [22].

Barber and Fox call such missed opportunities examples of negative serendipity [21].

### The role of background knowledge

The radiologist sees structures in a chest X-ray that the untrained eye does not notice. The quote by Pasteur at the beginning of this article that in the observational sciences chance favours only prepared minds, refers to the phenomenon that one must have the relevant background knowledge to notice and be able to interpret an unexpected finding. To see the unexpected, one obviously must know what to expect. Observations are not always objective and theory-neutral facts. Thomas Kuhn argues that what scientists perceive is, at least partly, determined by their beliefs. Observations are ‘theory loaded;’ they depend on knowledge of the field [23]. Physicists, for instance, failed to notice clearly visible tracks in cloud chambers caused by positrons before these particles were postulated by Paul Dirac in 1928 [24].

This view of serendipity fits well with Walpole’s definition stressing the importance of the combination of accident and sagacity. Interestingly, the three princes of Serendip, protagonists of the Persian fairy tale that Walpole refers to, were sophisticated hunters, and well-educated and trained in the art of tracking. Louis Liebenberg’s hypothesis is that the art of tracking was even the origin of science, a skill emerging two million years ago [25].

## Conclusion

ORL, like other domains of the medical sciences, is partly shaped by serendipitous findings of its contributing scientists. The eight case studies presented here are by no means isolated chance events, but possibly part of the experiences of every serious innovator. Its frequent occurrence does, however, not imply that a serendipitous finding can be predicted or consciously promoted. Its emergence is by definition unanticipated. Serendipity does, however, not emerge in an intellectual vacuum. As Pasteur remarked, chance favours only prepared minds.

## Data Availability

No data were used.

## Footnotes

1 Les observations médicales nouvelles se font généralement par hasard (…) L’initiative du médecin consiste à voir et à ne pas laisser échapper le fait que le hasard lui a offert, et son mérite se réduit à l’observer avec exactitude (…) Il n’y a rien d’accidentel, et ce qui pour nous est accident n’est qu’un fait inconnu qui peut devenir, si on l’explique, l’occasion d’une découverte plus ou moins importante [1].

2 (…) (par hasard diriez-vous peut-être, mais souvenez-vous que dans les sciences de l’observation le hasard ne favorise que des esprits préparés). Cited in [2].

3 A highly readable account of serendipitous discoveries in the field of ophthalmology already exists [4].

4 The classical example of serendipity in response to a perceived problem is of course the discovery of penicillin. Alexander Fleming was already looking for what we now call an antibiotic and published about one: lysozyme. Then he accidentally stumbled over an even more effective substance.

## References

[1] Bernard C (1943). Introduction à l’étude de la médecine expérimentale.

[2] Van Andel P, Bourcier D (2009) De la sérendipité dans la science, la technique, l’art et le droit. Leçons de l’inattendu. L’actmem, Chambéry.

[3] Van Andel P (1994). Anatomy of the unsought finding. Serendipity: Orgin, history, domains, traditions, appearances, patterns and programmability. Br J Philos Sci.

[4] Givner I, Lachterman A (1967). Serendipity in ophthalmology. Am J Ophthalmol.

[5] Silver S (2015). The prehistory of serendipity, from Bacon to Walpole. Isis.

[6] Merton RK, Barber E (2011). The travels and adventures of serendipity: a study in sociological semantics and the sociology of science.

[7] Reichenbach H (1938). Experience and prediction: an analysis of the foundations and the structure of knowledge.

[8] Popper KR (1979). Objective knowledge: an evolutionary approach.

[9] Garcia M (1881) On the invention of the laryngoscope. In: Transactions of the Seventh Section of the Laryngology of the VIIth International Congress of Medicine. 3,197. J. W. Kolckmann, London

[10] Feldmann H (2003). Bilder aus der Geschichte der Hals-Nasen-Ohren-Heilkunde.

[11] Huizinga E (1969). On esophagoscopy and sword-swallowing. Ann Otol Rhinol Laryngol.

[12] Czermák JN (1863). Der Kehlkopfspiegel: und seine Verwerthung für Physiologie und Medizin: Eine Monographie.

[13] Weir N (1990). Otolaryngology: an illustrated history.

[14] Meyer W (1870). On adenoid vegetations in the naso-pharyngeal cavity: their pathology, diagnosis, and treatment. Medicochirur Trans.

[15] Flourens MJP (1830). Expériences sur les canaux semi-circularies de l’oreille. Mém Acad Sci.

[16] Bárány R, Foundation N (1967). Some new methods for functional testing of the vestibular apparatus and the cerebellum. Nobel lectures, physiology or medicine 1901–1921.

[17] Poelmans J, Tack J, Feenstra L (2001). Chronic middle ear disease and gastroesophageal reflux disease: a causal relation?. Otol Neurotol.

[18] Poelmans J, Tack J, Feenstra L (2002). Prospective study on the incidence of chronic ear complaints related to gastroesophageal reflux and on the outcome of antireflux therapy. Ann Otol Rhinol Laryngol.

[19] Lapeña JF (2013). Mirrors and reflections: the evolution of indirect laryngoscopy. Ann Saudi Med.

[20] Clerf LH (1956). Manuel Garcia's contribution to laryngology. Bull N Y Acad Med.

[21] Barber B, Fox RC (1958). The case of the floppy-eared rabbits: an instance of serendipity gained and serendipity lost. Am J Sociol.

[22] Sleigh MA (1981). Primary ciliary dyskinesia. Lancet.

[23] Kuhn TS (1962). The structure of scientific revolutions.

[24] Hanson NR (1958). Patterns of discovery.

[25] Liebenberg L (2001). The art of tracking: the origin of science.

